# Brain Abscess Caused by Streptococcus intermedius and Aggregatibacter aphrophilus Secondary to Exfoliation of a Deciduous Tooth in a Previously Healthy Child: A Case Report

**DOI:** 10.7759/cureus.80591

**Published:** 2025-03-14

**Authors:** Kasumi Noda, Hiroki Miura, Kei Kozawa, Jun Muto, Tetsushi Yoshikawa

**Affiliations:** 1 Department of Pediatrics, Fujita Health University School of Medicine, Toyoake, JPN; 2 Department of Neurosurgery, Fujita Health University School of Medicine, Toyoake, JPN

**Keywords:** aggregatibacter aphrophilus, brain abscess, ring-enhancing brain lesion, streptococcus intermedius, tooth exfoliation

## Abstract

This report describes the case of a previously healthy nine-year-old girl who developed a left temporal lobe brain abscess following spontaneous exfoliation of a healthy deciduous tooth. She presented in convulsive status epilepticus without fever or focal neurological symptoms, and the initial MRI revealed a ring-enhancing lesion with adjacent dural inflammation. Surgical drainage and intravenous antibiotic therapy led to a full recovery with no neurological sequelae after six weeks of treatment. Pus culture revealed *Streptococcus intermedius* and *Aggregatibacter aphrophilus*, both of which are part of the normal oral flora. The route of infection in this case is suggested to be hematogenous spread from the site of tooth exfoliation, as evidenced by the ipsilateral location of the abscess. This highlights the potential for serious intracranial infections to originate from the exfoliation of a deciduous tooth in healthy children, particularly those with orthodontic appliances.

## Introduction

Brain abscess is a life-threatening infection, with a mortality rate of 20% [1]. Important risk factors include contiguous infection, neurosurgery, congenital heart disease, and immunocompromised host [1,2]. Intraoral foci of infection originating from the teeth or periodontium are also important causes of brain abscesses. Recently, the proportion of odontogenic brain abscesses has been shown to be higher than previously known, which might be due to increased awareness that oral infections can spread to distant anatomical sites [3,4]. The increasing frequency of odontogenic infection might be due to a larger elderly population vulnerable to infection, increased awareness that oral infections can spread to distant anatomical sites, and the possibility that odontogenic predisposing infections might have been previously overlooked [4]. Most reports of odontogenic brain abscesses have been in adults, and cases in children are rare; therefore, the risk factors in children remain unknown [4,5]. Here, we describe a case of a previously healthy child who developed a brain abscess after exfoliation of a healthy deciduous tooth. Her parents provided consent for this case report.

## Case presentation

A previously healthy nine-year-old girl was transferred to the emergency department in convulsive status epilepticus (Day 1). Although she had been lethargic five days prior to the hospital visit, there were no other signs suggesting central nervous system infection. She exhibited a decreased level of consciousness but did not have fever, neck stiffness, or focal neurological symptoms. She had no medical history suggestive of immunodeficiency and her immunizations were up-to-date. She had no known cardiac disease. Removable orthodontic appliances had been attached to her upper and lower teeth one year before the onset of the present illness, but routine dental examination had not revealed any problems with her oral hygiene, such as plaque buildup or gingivitis. Two weeks before the presentation, she experienced spontaneous exfoliation of the deciduous first molar on the left lower jaw.

Blood culture on admission was negative, but pus culture grew *Streptococcus intermedius* and *Aggregatibacter aphrophilus*. The patient underwent extensive screening for other potential sources of infection. Echocardiography revealed a structurally normal heart without vegetation. Sinusitis, mastoiditis, and otitis media were excluded by the otolaryngologist. A dental examination including orthopantomography ruled out cavities, periodontitis, and pericoronitis. Immunologic tests during the convalescent period, including immunoglobulin concentrations, complement activity, B- and T-lymphocyte counts, and CD4+ and CD8+ cell populations, were normal.

**Figure 1:**
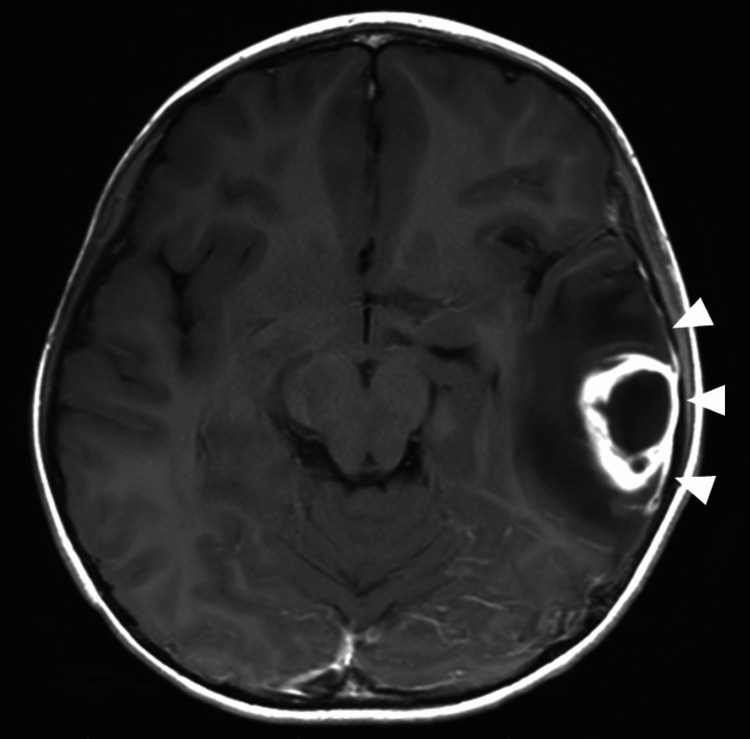
Magnetic resonance image of patient's brain. Axial T1-weighted magnetic resonance image with intravenous contrast showing a ring-enhancing abscess (32 mm × 24 mm) in the left temporal lobe associated with inflammation in the adjacent dura mater (white arrowheads).

Magnetic resonance imaging (MRI) revealed a ring-enhancing lesion in the left temporal lobe with adjacent dural inflammation (Figure 1). The abscess was surgically drained and she was treated with intravenous meropenem (135 mg/kg/day). Her condition improved rapidly, and she had a normal mental status by Day 2. After she completed six weeks of antibiotic treatment, she was discharged on Day 45 without any neurological sequelae. One and a half years after discharge, she has had no neurological sequelae including convulsions, and no abnormal EEG findings have been observed.

## Discussion

The most serious and fatal outcome of odontogenic infection is odontogenic brain abscess. Both *S. intermedius*, which is a β-hemolytic gram-positive member of the *Streptococcus anginosus* group (SAG), and *A. aphrophilus*, isolated from this patient, are part of normal oral flora and common causes of odontogenic brain abscesses. In a brain abscess, the most common route of infection has been a contiguous focus such as the middle ear, mastoid cells, or paranasal sinuses. However, it was recently demonstrated that 57% of brain abscesses are odontogenic, with SAG (88%) being the most commonly isolated bacteria, followed by* Fusobacterium nucleatum* (28%), *A. aphrophilus* (16%) [4]. Furthermore, as seen in the present case in which mixed infection with two types of bacteria was observed, odontogenic brain abscesses are more likely to involve multiple bacterial isolates, with 39-47% of cases isolating two or more bacterial species, compared to 23% in brain abscesses overall [1,6].

In patients with brain abscesses who do not have a contiguous focus of infection, such as our patient, the most likely source of infection is congenital heart disease, immunodeficiency, or dental infection [1-4]. Examinations revealed that our patient did not have congenital heart disease or immunodeficiency. It has been demonstrated that 40% of patients with intracranial bacterial infections of oral origin had intraoral symptoms or a dental procedure, with an average onset of symptoms 17.6 days after the procedure [6]. In our patient, a dental examination during hospitalization revealed no pathogenic oral conditions and the patient had no history of tooth extraction. Therefore, the most likely portal of entry for the bacteria was exfoliation of the deciduous first molar on the left lower jaw two weeks before illness onset. Thus, the route of bacterial infection in this case was thought to be hematogenous spread from the site of tooth exfoliation two weeks before the development of neurological symptoms. This hypothesis is also supported by the fact that the abscess was located in the left temporal lobe, ipsilateral to the site of tooth exfoliation. MRI finding of dural inflammation contiguous with the abscess also suggests hematogenous spread via the middle meningeal artery.

Odontogenic brain abscesses in children are extremely rare. According to previous literature, only 3-10% of brain abscess cases are attributed to odontogenic origins in children [7]. To the best of our knowledge, this is the first reported case of a brain abscess directly linked to spontaneous exfoliation of a healthy deciduous tooth, without recent dental procedure or other predisposing factors. A similar case has been reported in which a brain abscess developed four weeks after the application and tightening of braces; that case involved a dental procedure that may have directly contributed to the infection [8]. Meanwhile, our patient had no recent dental procedures, and the timing of the abscess strongly suggests hematogenous spread originating from the site of natural tooth exfoliation. Orthodontic appliances can significantly impact oral microbial changes and increase bacterial species, such as *Streptococcus* spp. [9]. In this patient, the timeline suggested the primary cause was the spontaneous exfoliation of a healthy deciduous tooth. However, the prolonged use of the orthodontic appliance may have indirectly contributed to bacterial colonization, creating an environment that facilitated the subsequent development of the brain abscess.

The American Heart Association and the American Dental Association recommend antibiotic prophylaxis for certain high-risk cardiac patients undergoing specific dental procedures to prevent infective endocarditis. However, these guidelines restrict antibiotic prophylaxis to procedures involving manipulation of the gingival tissue, periapical region of teeth, or perforation of the oral mucosa [10]. The present case suggests that the spontaneous exfoliation of a deciduous tooth can potentially lead to brain abscess in previously healthy children with orthodontic appliances, even in the absence of known risk factors.

## Conclusions

This case highlights the potential for serious intracranial infections, such as brain abscess, to arise from routine dental events like the spontaneous exfoliation of deciduous teeth, even in otherwise healthy children. Since the presence of orthodontic appliances significantly impact oral microbial changes and may increase the risk of such infections, underscoring the need for proper oral hygiene instruction and control. Further studies are warranted to evaluate the need for prophylactic antibiotics, particularly in patients using orthodontic appliances, to prevent severe outcomes.
